# Impact of the COVID-19 pandemic on pharmacy practice and on the provision of pharmaceutical care: A cross-sectional study among community pharmacists

**DOI:** 10.1177/27550834231161145

**Published:** 2023-03-31

**Authors:** Georges Hatem, Sara Ghamloush, Aya Al Chami, Mohammad Chaheen, Dalia Khachman, Sanaa Awada

**Affiliations:** 1 Clinical and Epidemiological Research Laboratory, Faculty of Pharmacy, Lebanese University, Beirut, Lebanon; 2 Faculty of Medicine, University of Porto, Porto, Portugal

**Keywords:** COVID-19 pandemic, community pharmacists, pharmacy practice, challenges, pharmaceutical care

## Abstract

**Background:**

Community pharmacists played an essential role in the control and management of the COVID-19 pandemic; both pharmaceutical care and community pharmacists were affected, given that, patients’ needs and demands increased due to the fear of lockdowns and shortage of medication throughout the pandemic.

**Objectives:**

This study was based in Lebanon and aimed to assess the impact of the COVID-19 pandemic on (1) pharmacists, including infection rates, pay, and working hours, and (2) pharmacy practice, including medicine and personal protective equipment (PPE) shortages.

**Design:**

A cross-sectional study involving 120 community pharmacists was carried out between August and November 2021.

**Methods:**

Data were collected using an online survey filled out by pharmacists working in Lebanon.

**Results:**

Most participants (71.7%) reported an increase in their income during the pandemic, and 60% reduced their working hours. A significant association was noted between being previously infected and marital status, level of education, work position, and salary of the participants. Most participants (95.8%) encountered a shortage of medications during the pandemic leading to high home storage of medication, searching for other sources of medicines, and decreased patient/pharmacist interactions.

**Conclusion:**

The COVID-19 pandemic imposed new challenges on pharmacists and the provision of pharmaceutical care. It affected pharmacists’ daily routines, putting them at risk of infection with limited availability of medicines and PPE. This study suggests that establishing effective crisis management plans to increase community pharmacists’ resilience during similar outbreaks.

## Introduction

In March 2020, a pneumonia outbreak was divulged as a pandemic by the World Health Organization (WHO) after the identification of its origin (severe acute respiratory syndrome coronavirus 2) and its global spread.^1^ Considering the fast transmission of the virus, the Centers for Disease Control and Prevention (CDC) recommended maintaining a physical distance of at least 1.8 m between people.^2^ Short-distance inhalation transmission can also occur, especially in crowded medical wards and inadequately ventilated spaces.^3^ The severity of cases was reported to be higher with age and smoking status and can sometimes lead to hospitalization or death.^4,5^ Nevertheless, people of all age groups with pre-existing comorbidities, such as diabetes, hypertension, cardiovascular diseases, chronic lung disease, immunosuppression, obesity, and cancer, had higher mortality rates.^5^

During the COVID-19 pandemic, community pharmacists faced several challenges due to the novelty of the virus and the lack of personal protective equipment (PPE); as a result, they have rearranged their workspaces and workflows to reduce the risk to their staff and patients.^6^ Furthermore, patients’ needs and demands increased due to the fear of lockdowns and shortage of medication.^7^ Moreover, supplies of PPE were scarce, considering the limited stock and inaptitude of fulfilling market requirements.^8^ To cope with this unpredictable state, several pharmaceutical companies implemented strict policies regarding drug supply and distribution channels to maintain patients’ health with narrowed stock.^9^ Some companies limited the allowed quantity to be ordered per month, which sometimes could not fulfill patients’ demands for chronic medications. Other companies applied a no-return policy of near-expiry or expired products and payments on a cash basis on receipt or a week after delivery.

Pharmacists worldwide tried to be resilient by playing a crucial role in managing and controlling the supply of medicines to the general population with a mandatory requirement of a medical prescription and patient identification card to order medicines. Frontline healthcare professionals, including pharmacists, interacted directly with patients suffering from financial, psychological, and health problems.^10^ Due to their work, many limited their working hours, stopped purchasing from certain pharmaceutical companies, or switched to generics while maintaining social distancing with their patients and limiting consultation time.^11^ Others followed the recommendations of the Ministry of Public Health in supporting medications manufactured locally while increasing the collaboration with physicians for the treatment options available.

Community pharmacists play an effective role in the management and control of the outbreak by promoting preventive strategies and maintaining their patients’ health.^12^ Given that they interacted with a large number of susceptible patients, they are indeed at higher risk of being infected by the virus.^13^ Community pharmacies in Lebanon are often the first point of care since pharmacists have extended roles in ensuring drug safety, counseling and prescribing some drugs, vaccination, patient education, and health promotion.^14^ The emergence of COVID-19 in February 2020 and the repetitive national lockdowns exacerbated the economic instability and unemployment rates.^15^ Consequently, pharmacists and pharmacy practice were affected because of the shortage of medications and access to PPE.^16^ Therefore, the objectives of this study were to assess the impact of the COVID-19 pandemic on pharmacists and pharmacy practice and evaluate the characteristics associated with previously infected pharmacists.

## Methods

### Study design

An observational cross-sectional study was carried out over 3 months between August and November 2021, targeting community pharmacists practicing throughout the pandemic. Data were collected using a survey developed after an extensive literature review.

### Study population and sample size calculation

Community pharmacists working during the pandemic were the main target population. They were visited at their site of work located in five governorates in Lebanon (Beirut, Mount Lebanon, North, South, and Bekaa) during pharmacies’ opening hours. An initial assessment was performed particularly to include those practicing during the pandemic to have a response with a greater fit to the study’s objectives. They were included based on pre-defined criteria, such as Lebanese pharmacists, workers in community pharmacies, and providers of signed informed consent. Those working in pharmaceutical companies were excluded from the study. As a result, 120 pharmacists participated in the study. After initial approval, an online questionnaire was sent to the included pharmacists.

Epi Info was used to calculate the required sample size using the following equation

$$N=\;\frac{{\left(Z_{1-\alpha /2}\right)}^{2}p(1-p)}{d^{2}}$$

where *Z* is a standard normal variate (*Z*_1−*α*/2_ = 1.96 at 95% confidence interval), *d* is the absolute accuracy or precision (9% margin of error), *P* is the expected proportion of the population with a specific outcome and was set at 0.5 (the recommended value if the proportion in the population is not known). According to the Order of Pharmacists in Lebanon report of 2019, 4113 community pharmacists were officially registered and consisted of the total target population.^17^ It was assumed that an increase in 20% occurred in the last 2 years. This yielded a required sample size of 119 participants. Power analysis (70.7%) was calculated using the G*Power software.

### Data collection

Data were collected using an online survey filled out by pharmacists (Supplemental material). The aforementioned survey was developed after a literature review taking into consideration two experts’ opinions. It included questions encompassing (1) the general characteristics of the participants (age, sex, governorate of work, marital status, level of education, work position, and monthly salary), (2) information regarding pharmacists’ assessment of the impact of COVID-19 on the pharmacist himself (being previously infected, source of infection, vaccination status, income, and working hours change) and on (3) pharmacy practice (patients’ flow, shortage of medication and its consequences, price, and shortage of PPE in addition to the main challenges faced). The survey was available in both English and Arabic based on participants’ preferences. It was disseminated online due to fear of long-term personal interviews during the COVID-19 outbreak and was filled at their time of preference to minimize recall bias.

### Ethical considerations

The study protocol, tool, and consent form were reviewed and approved by the institutional review board of the Faculty of Pharmacy of the Lebanese University (reference 7/21/D). Participants were informed about the study’s objective through a statement provided on the first page of the survey. On the same page, they were acknowledged that their participation was voluntary and that they could withdraw it at any point of the study with only provided answers registered. Consequently, written informed consent was provided by each participant. Anonymity and confidentiality were preserved since no name or personal data had been collected. Furthermore, no financial incentives were provided to the pharmacists. The protocol, data collection form, and dissemination of the results were considered for research purposes only.

### Statistical analysis

Statistical analyses were performed using Statistical Package for Social Sciences (SPSS Inc, Chicago, Illinois) Version 26. Categorical variables are presented using frequencies and percentages, and the hourly work rate is presented using mean and standard deviation. Bivariate analysis was conducted in which the dependent variable was the history of COVID-19 status (dichotomous) in association with the participant’s general characteristics. Chi-square or Fisher’s exact test was used to compare percentages between associate categorical variables. A *p*-value of less than 0.05 was considered statistically significant.

## Results

### General characteristics of the pharmacists

Overall, 256 potential participants were approached, of whom 120 (46.9% response rate) agreed to participate. The general characteristics of the sample are presented in Table 1. It consisted of 34.2% (*N* = 41) males and 65.8% (*N* = 79) females. Of these, 33 pharmacists (27.5%) were below 25 years of age, 56 (46.7%) were aged between 26 and 40 years, and 31 (25.8%) were aged above 40 years. Most of the sample had Mount Lebanon (36.7%) and Beirut (25.8%) as governorates of work. Almost 54% of the participants were married, with 31.7% having a bachelor’s degree in pharmacy practice and 60% with postgraduate studies. Pharmacists were equally distributed in terms of work positions, with the majority of employees working full-time (66.7%). The mean working rate per hour was 10.7 USD (*SD* = 5.1), with 63.4% of pharmacists earning more than 2000 USD monthly. At the time of the study, the official dollar rate was 1507 Lebanese Lira and 3500 in the black market.

**Table 1.** Distribution of the general characteristics of the pharmacists.

		Frequency (%)
Sex (*N* = 120)	Male	41 (34.2)
	Female	79 (65.8)
Age (years) (*N* = 120)	<25	33 (27.5)
	26–40	56 (46.7)
	>40	31 (25.8)
Governorate of work (*N* = 120)	Beirut	31 (25.8)
	Mount Lebanon	44 (36.7)
	North	21 (17.5)
	South	15 (12.5)
	Bekaa	9 (7.5)
Marital status (*N* = 120)	Single	55 (45.9)
	Married	65 (54.1)
Level of education (*N* = 120)	Pharmacy intern	10 (8.3)
	Graduate	38 (31.7)
	Postgraduate	72 (60)
Work position (*N* = 120)	Employee	60 (50)
	Pharmacy owner	60 (50)
Form of employment (*N* = 60)	Full-time	40 (66.7)
	Part-time	20 (33.3)
Rate per hour (USD) (*N* = 74)	*M* ± *SD*	10.7 ± 5.1
Salary per month (USD) (*N* = 120)	<1000	17 (14.1)
	1000–2000	27 (22.5)
	>2000	76 (63.4)

Results are given in terms of frequency (%); SD: standard deviation; USD: United States dollar.

### Impact of COVID-19 pandemic on pharmacists

Table 2 describes the impact of the COVID-19 pandemic on both pharmacists and pharmacy practice. On the individual level, 80 (66.7%) participants had a history of COVID-19 infection, out of which 16 (20%) reported their workplace as a source of infection and 24 (30%) did not know its origin. Most of the study sample (*N* = 86; 71.7%) had their income increased during the pandemic, and 72 (60%) reduced their working hours.

**Table 2.** Impact of COVID-19 pandemic on pharmacists and pharmacy practice.

Questions		Frequency (%)
Have you been infected with COVID-19? (*N* = 120)	Yes	80 (66.7)
	No	40 (33.3)
Have you got the infection from your workplace? (*N* = 80)	Yes	16 (20)
	No	40 (50)
	Don’t know	24 (30)
Have you been vaccinated for COVID-19? (*N* = 120)	Yes	112 (93.3)
	No	8 (6.7)
How was your income affected? (*N* = 120)	Increased	86 (71.7)
	The same	23 (19.1)
	Decreased	11 (9.2)
Did you reduce working hours during COVID-19? (*N* = 120)	Yes	72 (60)
	No	48 (40)

Results are given in terms of frequency (%).

Table 3 presents the bivariate analysis associating the status of COVID-19 infection with the general characteristics of the pharmacists. A statistically significant association with age groups was noted where 48.5% of pharmacists below 25 years were previously infected with the virus versus 75% and 71% of those between 26 and 40, and above 40 years, respectively, (*p* = 0.031). Moreover, 83.1% of married pharmacists were previously COVID-positive compared to only 47.3% of singles (*p* < 0.001). Level of education was significantly associated with the status of COVID-19 since half of the graduate pharmacists were previously positive compared to 76.4% of those with postgraduate studies (*p* = 0.018). A significantly higher percentage of pharmacy owners (85%) have been previously infected with the virus when compared to employees (48.3%) (*p* < 0.001). In addition, a higher percentage of pharmacists with a history of COVID-19 was noted per increase in monthly salary (*p* < 0.001).

**Table 3.** Association between being infected with COVID-19 and general characteristics of the pharmacists.

		Have you been infected with COVID-19?		
Variables		Yes	No	*p*
Sex (*N* = 120)	Male	28 (68.3)	13 (31.7)	0.785
	Female	52 (65.8)	27 (34.2)	
Age (years) (*N* = 120)	<25	16 (48.5)	17 (51.5)	**0.031**
	26–40	42 (75)	14 (25)	
	>40	22 (71)	9 (29)	
Marital status (*N* = 120)	Single	26 (47.3)	29 (52.7)	**<0.001**
	Married	54 (83.1)	11 (16.9)	
Level of education (*N* = 120)	Pharmacy intern	6 (60)	4 (40)	**0.018**
	Graduate	19 (50)	19 (50)	
	Postgraduate	55 (76.4)	17 (23.6)	
Work position (*N* = 120)	Employee	29 (48.3)	31 (51.7)	**<0.001**
	Pharmacy owner	51 (85)	9 (15)	
Form of employment (*N* = 60)	Full-time	20 (50)	20 (50)	0.715
	Part-time	9 (45)	11 (55)	
Salary per month (USD) (*N* = 120)	<1000	4 (23.5)	13 (76.5)	**<0.001**
	1000–2000	14 (51.9)	13 (48.1)	
	>2000	62 (81.6)	14 (18.4)	

USD: United States dollar.

Results are given in terms of frequency (%); chi-square or Fisher’s exact test (if less than 5 in a cell); *p-values* < 0.05 are presented in bold and are statistically significant.

### Impact of COVID-19 pandemic on pharmacy practice

Almost 96% outlined that the COVID-19 outbreak affected pharmacy practice with only 23.3% reporting a decrease in patient flow. Most of the participants (*N* = 115; 95.8%) encountered a shortage of medications during the pandemic. According to the sample, this shortage led to high home storage of medication (55%), getting medication from other sources (33.3%), and decreased patient/pharmacist interactions (11.7%). Moreover, only 19.2% of pharmacists experienced a shortage in PPE. Nonetheless, 94.2% reported increased prices (Table 4).

**Table 4.** Impact of COVID-19 pandemic on pharmacy practice.

Questions		Frequency (%)
Do you think COVID-19 impacted pharmacy practice? (*N* = 120)	Yes	115 (95.8)
	No	5 (4.2)
Did COVID-19 decrease patients’ flow? (*N* = 120)	Yes	28 (23.3)
	No	92 (76.7)
Did you encounter any shortage of medications? (*N* = 120)	Yes	115 (95.8)
	No	5 (4.2)
Do you think that shortage of medication led to: (*N* = 120)		
High home storage of medication		66 (55)
Lower patient/pharmacist relationship		14 (11.7)
Buying medication from other sources		40 (33.3)
Did you encounter shortage in protection equipment? (*N* = 120)	Yes	23 (19.2)
	No	97 (80.8)
Did the protection equipment price increase? (*N* = 120)	Yes	113 (94.2)
	No	7 (5.8)

**Figure 1.**
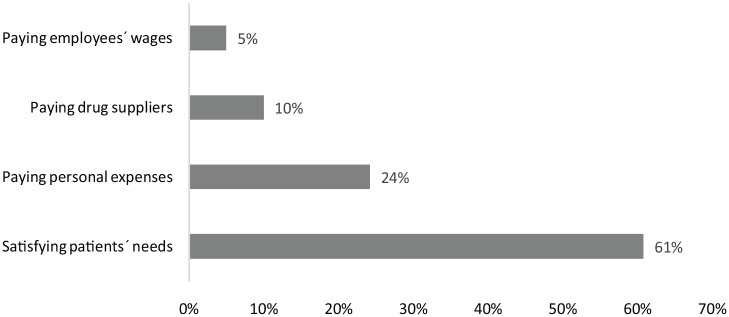
Reported challenges faced by pharmacists during the pandemic.

When asked about the main challenges encountered during the pandemic, more than half of the pharmacists (61%) considered the inability to satisfy patients’ needs as the main barrier. However, 24% reported difficulties in paying personal expenses, and 10% could not pay drug suppliers during that period (Figure 1).

## Discussion

Affecting several countries, the COVID-19 pandemic is considered a global public health issue due to the novelty of the virus and the rapidly increasing number of cases and mortality rate.^18^ The pandemic affected both healthcare and economic systems and impacted all health stakeholders, including physicians, pharmacists, patients, and insurers.^19^ This pilot study aimed to descriptively assess the impact of COVID-19 on community pharmacists and pharmacy practice in Lebanon.

Although an online survey was employed to improve dissemination, a satisfactory response rate was reported (46.9%),^20^ but significantly lower than the one reported among community pharmacists in the period before the pandemic.^21,22^ This can be related to the extension of the role of community pharmacists during the pandemic, such as authorization to prepare hand and surface disinfectants, promoting preventive measures to cope with the virus spread, and preparing compounding formulations in the pharmacy.^23^ Moreover, the supplementary workload to mitigate drug shortage, provide support in the therapeutic procedure of patients, and focus on those requiring professional attention with symptoms not related to the pandemic limited pharmacists’ access to social platforms.^24,25^ Other personal reasons could have affected the involvement of pharmacists, such as fear, anxiety, and lack of training on mental health and other challenges imposed by the COVID-19 outbreak.^21,26^

The study sample consisted predominantly of female pharmacists (65.8%) in agreement with the sex distribution of pharmacists according to the Order of Pharmacists in Lebanon database, wherein in 2018, 62.3% of registered pharmacists were women.^17^ A recent study in the United Kingdom reported that women were more likely to be involved in health protective behaviors and, therefore, could reflect the actual effect of the pandemic on both personal and professional advancement.^17^ Similar studies conducted to assess the impact of COVID-19 on pharmaceutical care services and community pharmacists in Ethiopia reported a majority of respondents within the age range of 26–35 years.^27^ In accordance, almost 47% of the pharmacists in this study were 26–40 years old. This age distribution could be related to the fact that the majority of the workforce in the pharmaceutical sector are young adults,^17^ and the higher susceptibility to being infected in the workplace with greater severity of infection in the older population.

The sample was equally distributed between pharmacy owners and employees. As a result, the perception of the impact of the COVID-19 pandemic could be investigated from two different points of view given that previous research conducted in Lebanon in 2017 outlined alterations in the financial profit and job satisfaction before the pandemic when taken from the perception of pharmacy owners.^28^ However, another study reported employees’ satisfaction in the pharmaceutical sector that contemplated patients’ satisfaction.^29^ Thus, the perception of the impact of COVID-19 might differ between pharmacy owners and employees.

Almost 67% reported being infected by the COVID-19 virus before the study time with only 20% delineating acquiring the infection from the workplace and 30% reporting an unknown source of infection. A multinational cross-sectional study in the Eastern Mediterranean countries highlighted community pharmacists’ higher susceptibility to acquiring the virus (12.9% versus 1.5% of the general population) which could be associated with encountering patients with limited PPE or even confirmed cases or colleagues.^30^ In addition, the increase in the number of patients and shortage of medication enhanced the physical contact with suspected cases, especially since home delivery services are illegitimate contrarily to other countries.^11^ Most participants (71.7%) reported an increase in their income during the pandemic, and 60% reduced their working hours. The economic crisis in Lebanon led to the devaluation of the currency and people’s purchasing power,^31^ which could have explained this high increase in income despite the decrease in shifts. Moreover, the limited human resources imposed the need for several pharmacists in the same shift and the performance of multi-tasks despite the earlier closure of pharmacies throughout the pandemic. Nonetheless, in this study, the size of the pharmacy was not considered, given that small community pharmacies could have accomplished higher profit in the first period of the pandemic but a potential loss of profit in a later stage due to their limited resources.^32^

The association between being previously infected by the COVID-19 virus and the general characteristics of the pharmacists might give a descriptive assessment of those more prone to be infected. Findings from this study showed a significant association with marital status (*p* < 0.001) with higher infectivity of married compared to single pharmacists. This can be explained by the fact that most probably single pharmacists live with their parents, who are at greater susceptibility and accordingly were adopting more strict measurements in their workplace. In agreement, a study conducted in Bangladesh in 2021 reported lower odds of good practice of PPE use among married healthcare workers when compared to unmarried ones.^33^ Furthermore, the level of education was significantly associated with COVID-19 status where a higher percentage was outlined in postgraduate pharmacists (76.4%; *p* = 0.018). A recent study found that pharmacists with postgraduate degrees had significantly higher perception scores toward the impact of their role and their involvement during the COVID-19 pandemic.^34^ In this study, those with a postgraduate degree had higher experience in clinical assessment and were more exposed to susceptible or confirmed cases than undergraduate and graduate pharmacists. In addition, the percentage of COVID-19 infected was significantly expanding per increase in pharmacists’ salaries in contrast with the findings of a retrospective cohort study carried out in Spain where workers with low salaries had a higher probability of COVID-19-confirmed infection.^35^ However, pharmacists with higher salaries might have worked more time and have had a higher number of patients, making them more prone to the virus. Their job description could also have covered broader aspects of pharmacy practice, such as blood pressure and glucose measurements, sales, and stock management.

Almost 96% of the pharmacists reported that the COVID-19 pandemic affected pharmacy practice. Among others, reports showed an increase in the role of community pharmacists in COVID-related counseling and patient education,^36^ namely due to the exhaustion of primary healthcare providers in Lebanon.^37^ Nevertheless, only a few physical interactions with patients existed and many common tasks were discontinued, such as blood pressure monitoring, glucose testing, and consultation groups.^36^ This perception was previously described in the literature where pharmacists reported drug scarcity and shortage in addition to spikes in prices of the available medications.^38,39^ However, 76.7% considered that the outbreak had not decreased the flow of patients in accordance with several reports highlighting an increasing number of people visiting community pharmacies due to their easier accessibility and involvement in the vaccination programs.^40,41^ Moreover, 55% of the participants reported that the medication shortage had led to high home storage of medication while 33% reported searching for other sources. These findings can be related to the fear of patients of not finding medicines and the ability of pharmacists to substitute to generics without referring to the physicians, conversely to previous restrictions related to the unified medical prescription implementation in Lebanon.^42,43^ In addition, despite the low prescription of generic medicines by Lebanese physicians, strategies toward encouraging them to prescribe generics can increase treatment options.^44^ When asked about the impact of the COVID-19 pandemic on PPE availability and price, pharmacists did not report any shortage, but an increase in their price. This impact was previously reported where the pandemic was accompanied by a serious increase in PPE use leading to spikes in their price.^45^

Finally, when asked about the main challenges faced during the pandemic, satisfying patients’ needs (61%) and paying personal expenses (24%) were the most reported challenges. Pharmacists were adopting different strategies, including dispensing medications in advance to minimize wait times and duplicate visits in addition to anticipatory management of medication-related needs.^46^ Furthermore, the pandemic imposed supplementary costs to provide protective measures, such as the use of masks and gloves by pharmacy staff, home services, and the provision of disinfectants.^47^

This study has several limitations. The sample size, contextual factors, and sociocultural characteristics in Lebanon might affect its external validity;^48^ therefore, its findings cannot be generalized to pharmacists in other settings. Among others, the culture in Lebanon can be different from other countries; since, despite that by law, pharmacists cannot prescribe medicines, such as antibiotics, research showed that they practically did it without referring patients to doctors.^49^ Purchasing power and out-of-pocket drug expenditures can also affect pharmacists’ roles and responses to patients’ needs.^37^ Using online platforms might have induced selection bias since mostly motivated pharmacists are willing to participate. However, this method eliminated interviewer biases and was considered more appropriate due to the strict measures related to the pandemic. Moreover, the concurrent occurrence of the economic crisis in Lebanon might have induced some bias related to its possible impact on both pharmacists and pharmacy practice suggesting a possible overestimation of the perceived impact of the pandemic. Nevertheless, to our knowledge, this is the first study tackling the impact of COVID-19 on community pharmacists and the pharmacy profession in Lebanon and, as a result, could provide a descriptive approach to the actual status of pharmacists and hence, better control. Furthermore, results from this study are propounding for a longitudinal study performed on a bigger sample size and allow for better control for confounders.

## Conclusion

The COVID-19 pandemic has affected community pharmacists worldwide, particularly in Lebanon, given the high workload and the improvised social and health consequences. The outbreak enforced several challenges on the personal and professional levels of pharmacy practice. Any progress toward healthcare sustainability requires learning from other nations about reforming the foundations of the healthcare system to establish effective crisis management plans and increase pharmacists’ resilience. Further investigation should be carried out, targeting a bigger sample size and focusing on the different boundaries that pharmacists faced throughout the pandemic.

## Supplemental Material

sj-docx-1-map-10.1177_27550834231161145 – Supplemental material for Impact of the COVID-19 pandemic on pharmacy practice and on the provision of pharmaceutical care: A cross-sectional study among community pharmacists Click here for additional data file. Supplemental material, sj-docx-1-map-10.1177_27550834231161145 for Impact of the COVID-19 pandemic on pharmacy practice and on the provision of pharmaceutical care: A cross-sectional study among community pharmacists by Georges Hatem, Sara Ghamloush, Aya Al Chami, Mohammad Chaheen, Dalia Khachman and Sanaa Awada in The Journal of Medicine Access

sj-pdf-2-map-10.1177_27550834231161145 – Supplemental material for Impact of the COVID-19 pandemic on pharmacy practice and on the provision of pharmaceutical care: A cross-sectional study among community pharmacists Click here for additional data file. Supplemental material, sj-pdf-2-map-10.1177_27550834231161145 for Impact of the COVID-19 pandemic on pharmacy practice and on the provision of pharmaceutical care: A cross-sectional study among community pharmacists by Georges Hatem, Sara Ghamloush, Aya Al Chami, Mohammad Chaheen, Dalia Khachman and Sanaa Awada in The Journal of Medicine Access

## Declarations

**Ethical approval and consent to participate:** After reviewing the study protocol, questionnaire, and consent form, the institutional review board of the Faculty of Pharmacy of the Lebanese University approved the study (reference 7/21/D). Written informed consent was obtained from each participant.

**Consent for publication:** Not applicable.

**Availability of data and materials:** Not applicable.
