# Supplementary material for: Impact of the COVID-19 pandemic on pharmacy practice and on the provision of pharmaceutical care: A cross-sectional study among community pharmacists
Source: J Med Access. 2023 Mar 31;7:27550834231161145. doi: 10.1177/27550834231161145 (PMC10067468; doi:10.1177/27550834231161145)
Supplement: sj-pdf-2-map-10.1177_27550834231161145 – Supplemental material for Impact of the COVID-19 pandemic on pharmacy practice and on the provision of pharmaceutical care: A cross-sectional study among community pharmacists [file sj-pdf-2-map-10.1177_27550834231161145.pdf]

Questionnaire

كما نعلم جميعًا ، اجتاحت كوفد-١٩ العالم .لقد نجحت في إغلاق العالم بأسره :من ناحية التباعد الاجتماعي وعمليات الإغلاق شائعة في جميع البلدان .يعاني الاقتصاد البناني منذ بداية عام 2019 ، وقد دُفع الآن إلى مستويات لم نشهدها من قبل.

•تهدف هذه الدراسة إلى تحليل تأثير كوفد-١٩ بالإضافة إلى الأزمات الاقتصادية السائدة على صيادلة المجتمع اللبناني ، واستجابتهم لهذا الوباء ، والتحديات التي واجهوها خلال هذه الفترة.

شكرًا لك على موافقتك على المشاركة في هذا الاستبيان .جميع إجاباتك خاصة وسرية ولن تستخدم إلا لأغراض البحث.

- *As we all know, COVID-19 has taken the world by storm. It has managed to shut down the entire world: social distancing and lock downs were common in all countries. In Lebanon, the economy has been suffering since the beginning of 2019 and now has been pushed to extents previously unseen.*
- *This study aims to analyze the impact of COVID-19 as well as the prevalent economic crises on the Lebanese community pharmacists, their response to COVID-19, and the challenges they faced during this period.*
- *Thank you for agreeing to take part in this survey. All of your answers are private and confidential and will only be used for research purposes.*

❖ ***Sociodemographic Information***

1. **Age** *(years)/* **العمر:**	☐ 18-25 ☐ 25-40 ☐ 40-60 ☐ >60
**2. Gender/ الجنس:**	☐ Male/ ذكر ☐ Female/ أنثى
**3. Education level/ المستوى التعليمي**	☐ Pharmacy student/ طالب صيدلي ☐ Graduate/ متخرج ☐ Post graduate/ دراسات عليا
**4. Marital status/ الحاله الإجتماعية:**	☐ Single/ أعزب ☐ Engaged / خاطب ☐ Married / متزوج ☐ Other/ غيره
**5. Nationality/الجنسية:**	☐ Lebanese/ لبناني ☐ Non-Lebanese/ غير لبناني
**6. Governorates/المنطقة:**	☐ Akkar/ عكار ☐ North/ الشمال ☐ Mount Lebanon/ جبل لبنان ☐ Beirut/ بيروت ☐ Beqaa/ البقاع ☐ Baalbek-Hermel/ بعلبك/الهرمل ☐ Nabatiye/ النبطية ☐ South Lebanon/ جنوب لبنان
**7. Workplace/ مكان العمل:**	☐ Akkar / عكار ☐ North/ الشمال ☐ Mount Lebanon/ جبل لبنان ☐ Beirut/ بيروت ☐ Bekaa / البقاع ☐ Baalbek-Hermel/ بعلبك/الهرمل ☐ Nabatiye/ النبطية ☐ South Lebanon/ جنوب لبنان
**8. Work status/ حالة العمل:**	☐ Pharmacy owner/ صاحب صيدلية ☐ Pharmacy employee/ full time/ موظف صيدليه / بدوام كامل ☐ Pharmacy employee/ part time/ موظف صيدلية / دوام جزئي ☐ Student/ part time/ طالب / دوام جزئي
**9. Salary range/ معدل المعاش:**	☐ <675,000 L.L<br>☐ 675,000 L.L - 1,499,000 L.L<br>☐ 1,500,000 – 2,999,000 L.L<br>☐ > 3,000,000 L.L<br>Please specify your hourly rate/ يرجى تحديد سعر الساعة الخاص بك : ………

PART 1: COVID-19 in Lebanon and its impact on the community pharmacists/
المجتمع صيادلة على وأثره لبنان ١٩في-كوفد

10. Have you been infected with COVID-19? /أصبت هل بفيروس كوفيد 19؟
☐ Yes /نعم
☐ No/كلا
☐ Not sure, didn't do the test/ لست متأكدا ، لم أقم بإجراء الاختبار

11. If you have been infected with covid-19 virus, did this infection was caused by a patient came to the pharmacy?/ إذا كنت مصابًا بفيروس كوفيد -19 ، فهل سبب هذه العدوى هو أن مريض جاء إلى الصيدلية؟
☐ Yes/نعم
☐ No/كلا
☐ I don't know/لا أعلم

12. Did you take the COVID-19 vaccine? If yes, which one did you take? هل تلقيت لقاح كوفد-19؟ إذا كانت الإجابة بنعم ، فماذا أخذت
☐ Yes/نعم: ……… ☐ No, not willing to/ كلا ، لا ارغب في ذلك
☐ No, but willing to / كلا ، لكن ارغب في ذلك

13. Do you think COVID-19 had brought an impact on the pharmaceutical care service? هل تعتقد أن كوفد-١٩ كان له تأثير على خدمة الرعاية الصيدلانية؟
☐ Yes /نعم
☐ No/كلا
Justify/برر جوابك:
……………………………………………………………………………………
……………………….

14. Do you think COVID-19 has decreased the patient flow rate to seek pharmaceutical care? هل تعتقد أن كوفد-١٩ قد قلل من معدل تدفق المرضى للحصول على الرعاية الصيدلانية؟
☐ Yes /نعم ☐ No /كلا

15. What are the most sold medications/para-medicals during this pandemic? ما هي الأدوية / شبه الطبية الأكثر مبيعًا خلال هذه الجائحة
☐ Masks/أقنعة
☐ Vitamin C/ zinc/فيتامين C و زنك
☐ Panadol/بندول
☐ hygiene and alcohol/معقم يدين و سبيرتو
☐ antibiotics/ مضادات حيوية
☐ antivirals/مضادات الفيروسات
☐ others/غيره: ……..

16. Did you encounter any shortage of pharmaceutical medicines because of the pandemic? هل واجهت أي نقص في الأدوية الصيدلانية بسبب الوباء
☐ Yes/نعم ☐ No/كلا
Name the main COVID-19 medicines that were out of stock during this pandemic/ قم بتسمية أدوية كوفد-١٩ الرئيسية التي نفد مخزونها خلال هذه الجائحة

17. Did you encounter any shortage of personal protective equipment (PPE's) and the personal hygiene material because of the pandemic? هل واجهت أي نقص في معدات الحماية الشخصية (PPE's) ومواد النظافة الشخصية بسبب الوباء
☐ Yes / نعم ☐ No /كلا

18. Did you encounter any change in the cost of PPE's and the pharmaceutical medicines used in COVID-19 (VITAMIN C, ZINC, VITAMIN D…)? because of the pandemic? هل واجهت أي تغيير في تكلفة معدات الوقاية الشخصية والأدوية الصيدلانية المستخدمة في كوفد-١٩(VITAMIN C ، ZINC ، VITAMIN D ...)؟ بسبب الوباء؟

 Yes /نعم No /كلا

If yes, please describe how:/ إذا كانت الإجابة بنعم ، يرجى وصف كيفية القيام بذلك:

 decreased from usual/ انخفض عن المعتاد increased by <50% / زاد بمعدل

 increased by 50-100% / زاد بمعدل increased by >100%// زاد بمعدل

19. Did you had to shut down your pharmacy during this pandemic? هل اضطررت إلى إغلاق صيدليتك أثناء هذا الوباء؟

 Yes/نعم No/كلا No, but I reduced working hours/ لا ، لكني خفضت ساعات العمل

Do you agree or disagree with the following statements? هل توافق أو لا توافق على العبارات التالية؟

20. The Ministry of Public Health in Lebanon stood beside community pharmacists during this pandemic / وقفت وزارة الصحة العامة في لبنان إلى جانب صيادلة المجتمع خلال هذا الوباء

 Strongly disagree / اعارض بشدة Disagree/ اعارض Neither / لا أوافق ولا اعارض Agree / أوافق

 Strongly Agree / أوافق بشدة

21. The MOPH in Lebanon organized series of webinars to ensure pharmacists' updated knowledge towards COVID-19/ نظمت وزارة الصحة العامة في لبنان سلسلة من الندوات عبر الإنترنت لضمان معرفة الصيادلة بـ كوفد-١٩ المحدثة

 Strongly disagree/ اعارض بشدة Disagree/ اعارض Neither/ لا أوافق ولا اعارض Agree / أوافق

 Strongly Agree/ أوافق بشدة

22. Patient – Pharmacist relationship were strengthened during this pandemic/ تم تعزيز العلاقة بين المريض والصيدلاني خلال هذه الجائحة

 Strongly disagree / اعارض بشدة Disagree / اعارض Neither/ لا أوافق ولا اعارض Agree

 Strongly Agree/ أوافق بشدة

23. The Order of Pharmacists in Lebanon (OPL) stood beside community pharmacists during this pandemic and took important actions to mitigate the severity of this pandemic on the pharmacists/ وقفت نقابة الصيادلة في لبنان إلى جانب صيادلة المجتمع خلال هذا الوباء واتخذت إجراءات مهمة للتخفيف من حدة هذا الوباء على الصيادلة

 Strongly disagree/ اعارض بشدة Disagree/ اعارض Neither/ لا أوافق ولا اعارض

 Agree/ أوافق

 Strongly Agree/ أوافق بشدة

PART 2: Impact of the Lebanese economic crisis on the community pharmacists/ المجتمع صيادلة على اللبنانية الاقتصادية الأزمة تأثير

24. How did your salary change during this economic crisis? الأزمة هذه خلال راتبك تغير كيف الاقتصادية؟

☐ increased/ إرتفع ☐ decreased/ إنخفض ☐ still the same/ يتغير لم

25. Are you satisfied with this change in your salary? Does it cover all your expenses? هل نفقاتك؟ كل يغطي هل راتبك؟ في التغيير هذا عن راض أنت

☐ No/ كلا: ………………………………….

☐ Yes/ نعم: ……………………………………

26. What was the main problem you were suffering from because of this financial crisis? المالية؟ الأزمة هذه بسبب منها تعاني كنت التي الرئيسية المشكلة هي ما

☐ Struggle to pay for suppliers/ للموردين الدفع أجل من صعوبة

☐ Struggle to pay employee's salary/ الموظف راتب دفع أجل من صعوبة

☐ Struggle to pay your expenses/ نفقاتك لدفع صعوبة

☐ Struggle to dispense patients' medications/ المرضى أدوية صرف أجل من صعوبة

27. Which category of drug was the most out of stock? من نفادًا الأكثر كانت الأدوية من فئة أي المخزون؟

☐ chronic medications/ مزمنة أدوية ☐ Para-pharmaceutical/ صيدلاني شبه

☐ over the counter (OTC) medications/ طبية وصفة تستلزم لا التي الأدوية ☐ Others/ غيره:

………

28. Please name some (two or three) chronic medications and OTC that are still unavailable والتي طبية وصفة تتطلب لا التي والأدوية المزمنة الأدوية من (ثلاثة أو اثنين) بعض تسمية يرجى متوفرة غير تزال لا

☐ Chronic/مزمن: …………………………………………

☐ OTC/ طبية وصفة بدون دواء: …………………………………………………

29. This shortage in chronic and OTC drug have led to: والأدوية المزمنة الأدوية في النقص هذا أدى إلى طبية وصفة تستلزم لا التي:

☐ Patients are buying their medicines from outside/ الخارج من أدويتهم يشترون المرضى

☐ Patients are storing a big quantity of their medicines/ أدويتهم من كبيرة كمية بتخزين المرضى يقوم

☐ Patient – Pharmacist relationship has been shaken/ والصيدلاني المريض بين العلاقة اهتزت

30. This huge increase in US dollar versus Lebanese Lira affected you by/ الهائلة الزيادة هذه خلال من بها تأثرت اللبنانية الليرة أمام الأمريكي الدولار قيمة في

☐ Loss of your stock/ مخزونك فقدان

☐ Huge material loss/ ضخمة مادية خسارة

☐ Inability to buy para-pharmaceutical/ الصيدلانية شبه الأدوية شراء على القدرة عدم

☐ Inability to afford expenses/ النفقات تحمل على القدرة عدم

☐ shut down your pharmacy/ بك الخاصة الصيدلية أغلق

31. How do you rate the attitude of the below pharmaceutical company in drug supply during this crisis? Abela, Benta, Catafago, Fattal, FDC, Mersaco, Omnipharma, Pharmaline, Sadco, Union/ الأزمة هذه خلال الأدوية توريد من أدناه الأدوية شركة موقف تقيم كيف

☐ Excellent/ ممتازة ☐ Very good/ جداً جيدة ☐ Good/ جيدة ☐ Bad/ سيئة ☐ Very Bad/ جداً سيئة

32. Many pharmaceutical companies have implemented strict conditions in drug supply these days. How did you deal with it?/ توريد في صارمة شروط الأدوية شركات من العديد طبقت الشروط؟ هذه مع تكيفت كيف .الأيام هذه خلال الأدوية

a- Supply a limited quantity (1 or 2 boxes) per month of main chronic drugs/ توريد الرئيسية المزمنة الأدوية من شهريًا (صندوق 2 أو 1) محدودة كمية

☐ limited the number of my patients/ حدت من عدد مرضاي

☐ dispense the chronic medicine every month for a different patient/ صرف الأدوية المزمنة كل شهر لمريض

☐ put a list of priorities for my patients/ وضع قائمة أولويات لمرضاي

☐ others/ غيره: ……………

b- Stop returning expired drugs or with near expiry date/ التوقف عن إعادة الأدوية منتهية الصلاحية أو التي قرب تاريخ انتهاء صلاحيتها

☐ contacted the company and stopped working with them/ اتصلت بالشركة وتوقفت عن العمل معهم

☐ made a group with other pharmacists to exchange patients and medicines/ كونت مجموعة مع صيادلة آخرين لتبادل المرضى والأدوية

☐ others/ غيره: ……………

c- Invoices must be paid on cash basis or after 1 week of delivery/ يجب دفع الفواتير نقدًا أو بعد أسبوع واحد من التسليم

☐ I stopped working with these companies/ توقفت عن العمل مع هذه الشركات

☐ I took a debt/ أخذت دين

☐ this condition didn't bother me at all/ هذا الوضع لم يزعجني على الإطلاق

d- The mandatory need of a prescription and the patient ID to order a medicine/ الحاجة الإلزامية إلى وصفة طبية وهوية المريض لطلب الدواء

☐ I stopped working with these companies/ توقفت عن العمل مع هذه الشركات

☐ I accepted this and ordered a prescription from all of my patients/ قبلت هذا وطلبت وصفة طبية من جميع مرضاي

☐ I switched my patients to a generic that is available/ لقد قمت بتحويل مرضاي إلى دواء بديل متوفر

33. Since October 2019, we all knew that a day will come and the Banque Du Liban would not be able to afford drug supply. This delay in proposing a law to rationalize drug subsidies had/ منذ تشرين الأول (أكتوبر) 2019 ، علمنا جميعًا أن يومًا سيأتي ولن يكون مصرف لبنان قادرًا على توفيرالأدوية. أدى هذا التأخير في اقتراح قانون لترشيد دعم الأدوية إلى:

☐ Encourage drug contempt and thus, drug shortage/ تشجيع إحتكار الدواء وبالتالي النقص فيه

☐ Encourage the patient to store his own medicine because of fear of its shortage/ تشجيع المريض على تخزين أدويته خوفا من نقصها

☐ Encourage the smuggling of medicine/ التشجيع على تهريب الأدوية

☐ Led to pharmacies bankrupt/ ادى الى افلاس الصيدليات

34. Are you satisfied with the actions taken by MOPH and OPL during this crisis?/ هل أنت راضٍ عن الإجراءات التي اتخذتها وزارة الصحة العامة و نقابة الصيادلة خلال هذه الأزمة

☐ No/كلا : ………………………………….

☐ Yes/نعم: ……………………………………

35. Did you believe that our government could handle this pandemic with economic crisis?/ هل تعتقد أن حكومتنا يمكن أن تتعامل مع هذا الوباء في هذه الأزمة الاقتصادية

☐ No/كلا

☐ Yes/نعم

36. What are your solutions for a better community pharmacy practice during this period?/ ما هي الحلول التي تقدمها لممارسة أفضل للقطاع الصيدلي خلال هذه الفترة؟

……………………………………………………………………………………………
………………..

……………………………………………………………………………………………
………………..

……………………………………………………………………………………………
………………..

……………………………………………………………………………………………
………………..

Thank you for your participation
